# Ultrasound properties of articular cartilage in the tibio-femoral joint in knee osteoarthritis: relation to clinical assessment (International Cartilage Repair Society grade)

**DOI:** 10.1186/ar2452

**Published:** 2008-07-13

**Authors:** Hiroshi Kuroki, Yasuaki Nakagawa, Koji Mori, Masahiko Kobayashi, Ko Yasura, Yukihiro Okamoto, Takashi Suzuki, Kohei Nishitani, Takashi Nakamura

**Affiliations:** 1 Department of Physical Therapy, Human Health Sciences, Graduate School of Medicine, Kyoto University, 53 Kawahara-Cho, Shogoin, Sakyo-Ku, Kyoto 606-8507, Japan; 2 Department of Orthopaedic Surgery, Graduate School of Medicine, Kyoto University, 54 Kawahara-Cho, Shogoin, Sakyo-Ku, Kyoto 606-8507, Japan; 3 Department of Applied Medical Engineering Science, Graduate School of Medicine, Yamaguchi University, 2-16-1 Tokiwadai, Ube, Yamaguchi 755-8611, Japan

## Abstract

**Introduction:**

There is a lack of data relating the macroscopic appearance of cartilage to its ultrasound properties. The purpose of the present study was to evaluate degenerated cartilage and healthy-looking cartilage using an ultrasound system.

**Methods:**

Ultrasound properties – signal intensity (a measure of superficial cartilage integrity), echo duration (a parameter related to the surface irregularity) and the interval between signals (that is, time of flight – which is related to the thickness and ultrasound speed of cartilage) – of 20 knees were measured at seven sites: the lateral femoral condyle (site A, anterior; site B, posterior), the medial condyle (site C), the lateral tibial plateau (site D, center; site E, under the meniscus) and the medial tibial plateau (site F, anterior; site G, posterior). The sites were evaluated macroscopically and classed using the International Cartilage Repair Society (ICRS) grading system.

**Results:**

The signal intensity of grade 0 cartilage was significantly greater than the intensities of grade 1, grade 2 or grade 3 cartilage. Signal intensity decreased with increasing ICRS grades. The signal intensity was greater at site B than at site C, site D, site F and site G. The signal intensity of grade 0 was greater at site B than at site E. The echo duration did not differ between the grades and between the sites. The interval between signals of grade 3 was less than the intervals of grade 0, grade 1 or grade 2. The interval between signals at site C was less than the intervals at site A, site B, site D, and site E.

**Conclusion:**

Site-specific differences in signal intensity suggest that a superficial collagen network may be maintained in cartilage of the lateral condyle but may deteriorate in cartilage of the medial condyle and the medial tibial plateau in varus knee osteoarthritis. Signal intensity may be helpful to differentiate ICRS grades, especially grade 0 cartilage from grade 1 cartilage.

## Introduction

Osteoarthritis is a degenerative disorder that progresses slowly, characterized by erosive deterioration of articular cartilage. Changes in the cartilage structure and composition, in morphologic and metabolic features, and in mechanical properties occur during the development and progression of osteoarthritis.

Studies using high-frequency pulse-echo ultrasound have elucidated several features of articular cartilage. Ultrasound may provide information about the integrity of cartilage [1-5] and the thickness of cartilage [1,6,7] by assuming a predefined ultrasound speed within the tissue, and ultrasound assessment of cartilage degeneration has been extensively studied [8-15]. Although it is believed that osteoarthritis begins with fibrillation of superficial cartilage and then progresses to the deep zone of cartilage, the very early events that occur on the surface of normal articular cartilage are unknown.

The International Cartilage Repair Society (ICRS) describes cartilage standard evaluation as follows: grade 0, normal cartilage; grade 1, near-normal cartilage with superficial lesions; grade 2, cartilage with lesions extending to <50% of the depth of the cartilage; grade 3, cartilage with defects that extend to >50% of the depth of the cartilage; and grade 4, severely abnormal cartilage in which the cartilage defects reach subchondral bone [16]. A study on the relationship between ICRS grades and mechanical properties of articular cartilage was reported recently [17]. The study mentioned that differentiating between healthy cartilage and ICRS grade 1 cartilage may be difficult using mechanical testing alone [17].

Ultrasound studies have revealed that high-frequency pulse-echo ultrasound is sensitive for detecting degeneration of the superficial collagen-rich cartilage zone [10], and that ultrasound detects microstructural changes up to a depth of 500 *μ*m [18]. Ultrasound measurements also appear to be related to changes in the extracellular matrix collagen and fibrillar network organization [12]. To our knowledge, there are no ultrasound studies on ICRS grades. The purpose of our study was therefore to investigate the relationship between ICRS grades and ultrasound properties. In addition, site-specific differences in the ultrasound properties of cartilage were investigated. We hypothesized that the ultrasound response of articular cartilage would be related to its ICRS grading.

## Methods

### Patients

From January 2003 to March 2004, patients with knee osteoarthritis who were attending the knee clinic at the Department of Orthopedic Surgery, Kyoto University Hospital, were screened for eligibility to undergo total knee arthroplasty. Patients who were diagnosed with varus knee osteoarthritis, common in Japan, underwent total knee arthroplasty and were involved in the present study. Twenty knees of 20 patients (mean age, 76 years; age range, 68 to 83 years; two males and 18 females) who gave informed consent to ultrasound measurement of their articular cartilages were studied. During the usual total knee arthroplasty procedure, after the knee joint was opened, ultrasound evaluation of articular cartilage was conducted at the femoral condyles and tibial plateaus *in vivo*. After ultrasound evaluation, the articular cartilages and bone were cut and trimmed for total knee arthroplasty.

**Figure 1:**
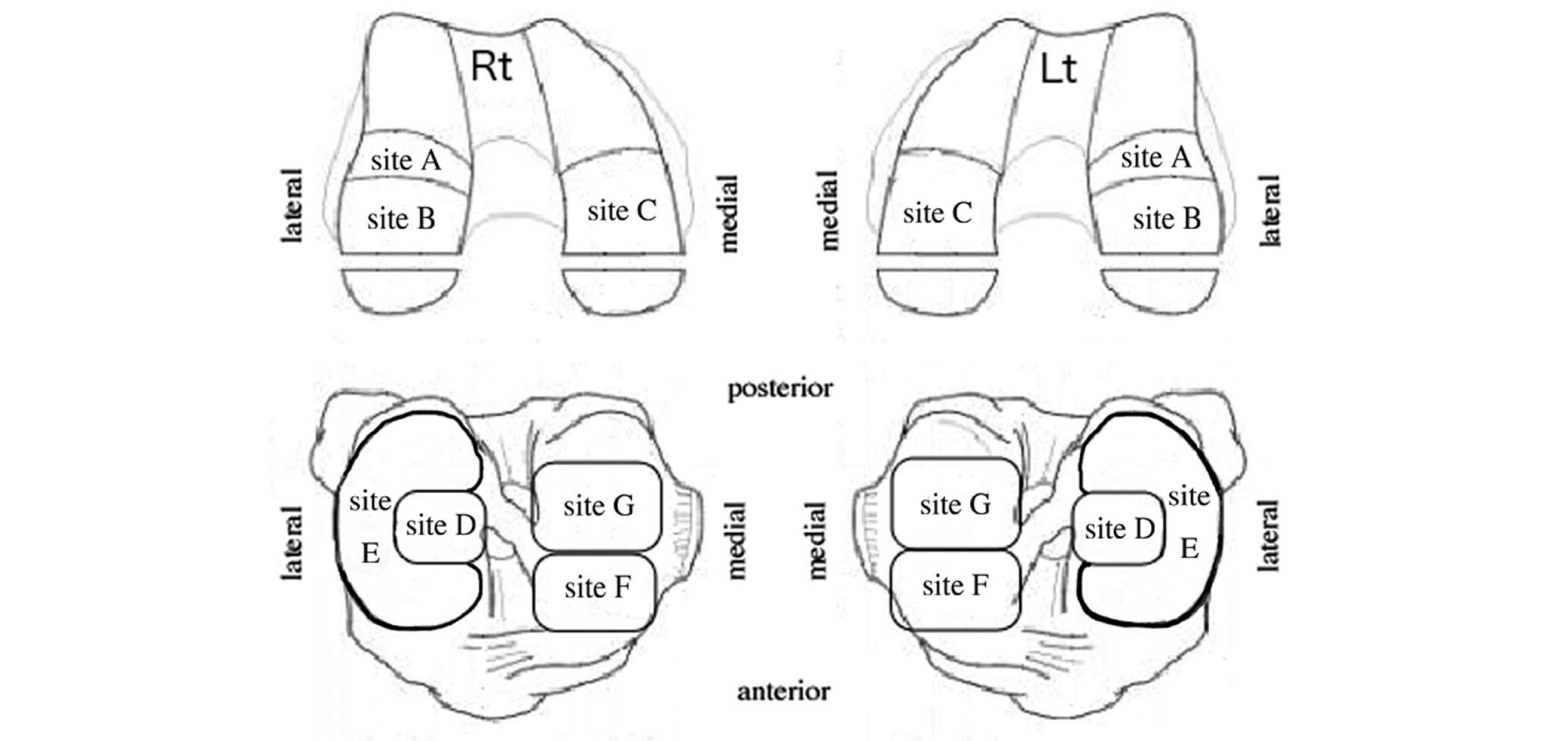
**Anatomical location of the knee**. Site A, femoral lateral condyle (anterior); site B, lateral condyle (posterior); site C, medial condyle; site D, lateral tibial plateau (center); site E, lateral tibial plateau (under the meniscus); site F, medial tibial plateau (anterior); site G, medial tibial plateau (posterior). Rt, right; Lt, left.

We modified the ICRS articular cartilage injury mapping system [16] and defined the seven sites of knee cartilage: site A, femoral lateral condyle (anterior); site B, lateral condyle (posterior); site C, medial condyle; site D, lateral tibial plateau (center); site E, lateral tibial plateau (under the meniscus); site F, medial tibial plateau (anterior); and site G, medial tibial plateau (posterior) (Figure 1).

### Ultrasound evaluation

Before ultrasound evaluation, cartilage at the seven sites was evaluated macroscopically using the ICRS articular cartilage injury classification system to determine the grade of severity of osteoarthritis. At least two surgeons joined in the macroscopic evaluation and agreed with the grading decision. After the grading had been made, the signal intensity (a measure of superficial cartilage integrity), the echo duration (a parameter related to the surface irregularity) and the interval between signals (that is, time of flight – which is related to thickness and ultrasound speed of cartilage) were measured using an ultrasound system that has been described previously [11,15,19].

**Figure 2:**
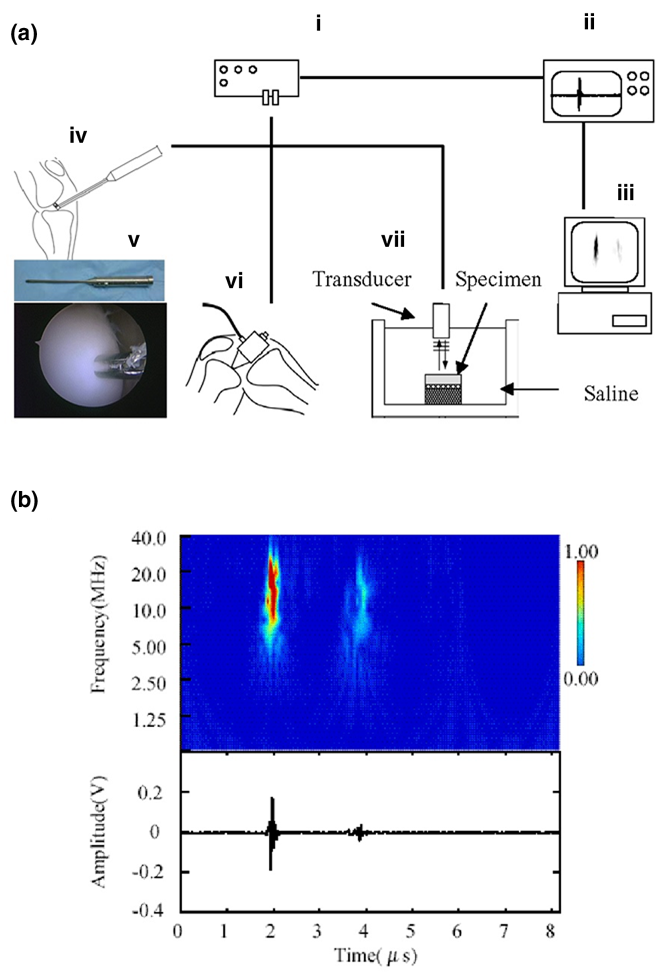
**The ultrasound system, typical ultrasound echo and wavelet map**. **(a) **The ultrasound measurement system employed, consisting of a transducer, a pulser/receiver (i), a digital oscilloscope (ii) and a personal computer (iii). The system can be used with arthroscopy (iv, v), open surgery (vi) or a saline bath (vii) for experimental measurement. The ultrasound wave output from the transducer travels through the saline. The reflected waves return to the transducer and generate electrical signals that are proportional to their intensity. **(b) **Typical ultrasound echo (lower) and wavelet map (upper). The wavelet map was calculated from the ultrasound echo using wavelet transform. The first (left) of the two large-amplitude groups was the echo (*t *= 2.0 μs: Group N) reflected from the cartilage surface, and the second (*t *= 3.9 μs: Group K) was reflected from the subchondral bone (right). The signal intensity (as shown by the scale) of Group N is a measure of superficial cartilage integrity. The time interval between Group N and Group K is related to thickness and ultrasound speed of cartilage. The echo duration of Group N is a parameter related to the surface irregularity of cartilage. See [20,30,31].

Briefly, the ultrasound system consists of a transducer, a pulser/receiver (Olympus NDT Japan Inc., Tokyo, Japan) and a personal computer, and provides a method for quantitatively evaluating articular cartilage (Figure 2a). The system can be set up for arthroscopic use, for open surgery, or with a saline bath for experimental measurement. The diameter of the transducer is approximately 3 mm and it is covered with a saline-filled cone.

For the present study, the ultrasound system was set at a 10 MHz center frequency, the sampling frequency was 500 MHz, no filtering or averaging was applied, and the system was set up for open surgery. The nominal center frequency of the transducers was 10 MHz (virtual center frequency, 12.6 MHz). The bandwidth at -6 dB was 7.7 to 17.4 MHz. The target was a 0.3175-cm diameter steel ball and the water path was 0.8509 centimeters, as per the manufacturer's instructions.

Using the wavelet transform for ultrasound reflection waves from the cartilage surface and from the subchondral bone [11,14,19], the three acoustic parameters (signal intensity, echo duration and interval between signals of cartilage) could be analyzed (Figure 2b). The wavelet transform is defined by the following equation:

$$W\left(a,b\right)=\frac{1}{\sqrt{a}}\int_{-\infty }^{\infty }f(t){\bar{\varphi }}_{a,b}\left(\frac{t-b}{a}\right)\text{d}t$$

where the function *f*(*t*) is the ultrasound wave. The function *φ*_*a*, *b*_(*t*) is the mother wavelet (${\bar{\varphi }}_{a,b}(t)$ is the complex conjugate of *φ*_*a*, *b*_(*t*)), where *a *is a dilation parameter and *b *is a translation parameter. In this system, we use the Gabor function as the mother wavelet. The equation is given by:

$$\varphi \left(t\right)=\frac{1}{\sqrt{\pi 4}}{\left(\frac{\omega_{p}}{\gamma }\right)}^{1/2}\exp\left\{-\frac{1}{2}{\left(\frac{\omega_{p}}{\gamma }t\right)}^{2}+i\omega_{p}t\right\}$$

where *ω*_*p *_is the center of frequency and *λ *is the frequency bandwidth.

In the present study, *ω*_*p *_was set at 40 MHz and *λ *was set at 5.336. The *λ *values were selected to approximately satisfy the Gabor function as $\gamma =\pi \sqrt{2/\ln2}\approx 5.336$ and can be used as the mother wavelet.

Three acoustic parameters were obtained from 510 points. A few measurements were conducted for each of the 510 measurement points, and finally the measurement in which the highest reflection wave from the cartilage surface was obtained was considered the acoustic parameter for each point – because the magnitude of signal intensity is greatest when the direction of the reflection wave is perpendicular. The same surgeon conducted all ultrasound measurements. Acoustic parameters from 38 points were not readable because the reflected ultrasound waves from the cartilage surface and from the subchondral bone overlapped and could not be differentiated. Mean values were calculated in cases where measurements were conducted in the same grades and in the same sites of the same knees. By this averaging procedure, 229 data for 20 knees were obtained from the 472 points (Additional file 1). Acoustic parameters from ICRS grade 4 tissues (68 data from 20 knees) were not used for the present study as, by definition, grade 4 tissue demonstrates full-thickness cartilage loss. The acoustic parameters obtained from the remaining 161 data sets of ICRS grade 0, grade 1, grade 2 and grade 3 tissues were therefore used for the study (Table 1). The data were blind-coded and analyzed by a researcher who is not a surgeon.

**Table 1:** Number of knees (percentage of 20 knees) at each site and at each grade

	Grade 0	Grade 1	Grade 2	Grade 3	Grade 4
Site A	11 (55)	12 (60)	6 (30)	0 (0)	4 (20)
Site B	16 (80)	8 (40)	5 (25)	0 (0)	3 (15)
Site C	0 (0)	0 (0)	1 (5)	13 (65)	20 (100)
Site D	1 (5)	11 (55)	10 (50)	1 (5)	3 (15)
Site E	17 (85)	7 (35)	1 (5)	0 (0)	1 (5)
Site F	0 (0)	0 (0)	3 (15)	15 (75)	18 (90)
Site G	0 (0)	2 (10)	11 (55)	10 (50)	19 (95)

For International Cartilage Repair Society grades, see Introduction. For anatomical location of sites, see Figure 1.

### Statistical analysis

Because the number of individual points measured varied between the 20 knees (Additional file 1), mean values were calculated for the individual knees if more than two points were measured at each grade and at each site. By this averaging, one datum per knee was allocated at each grade and at each site. Because 16 out of the 20 knees provided all the data from grade 0 to grade 3, the data of the grades from the 16 knees were compared statistically using the nonparametric Friedman test (*P *< 0.05 was taken as statistically significant). The *post hoc *Scheffe *F *test was used for multiple comparison among the grades. Because 11 out of the 20 knees had all the data of grade 0 cartilage at sites A, B and E, the signal intensity of the grade 0 cartilage of sites A, B and E was also compared in the 11 knees using the nonparametric Friedman test and the *post hoc *Scheffe *F *test.

Because 10 out of the 20 knees provided all the data from site A to site G, the data of the sites from the 10 knees were compared statistically using the nonparametric Friedman test (*P *< 0.05 was taken as statistically significant). The *post hoc *Scheffe *F *test was used for multiple comparison among the sites.

The coefficients of correlation of the three acoustic parameters, using test–retest reliability in 11 measurements, were 0.94 for the signal intensity, 0.78 for the echo duration and 0.99 for the interval between signals.

## Results

Of the ICRS grades, grade 0 cartilage comprised 55% (11 out of 20 knees), 80%, 5% and 85%, respectively, at site A, site B, site D and site E, and comprised 0% at sites C, F and G (Table 1).

**Figure 3:**
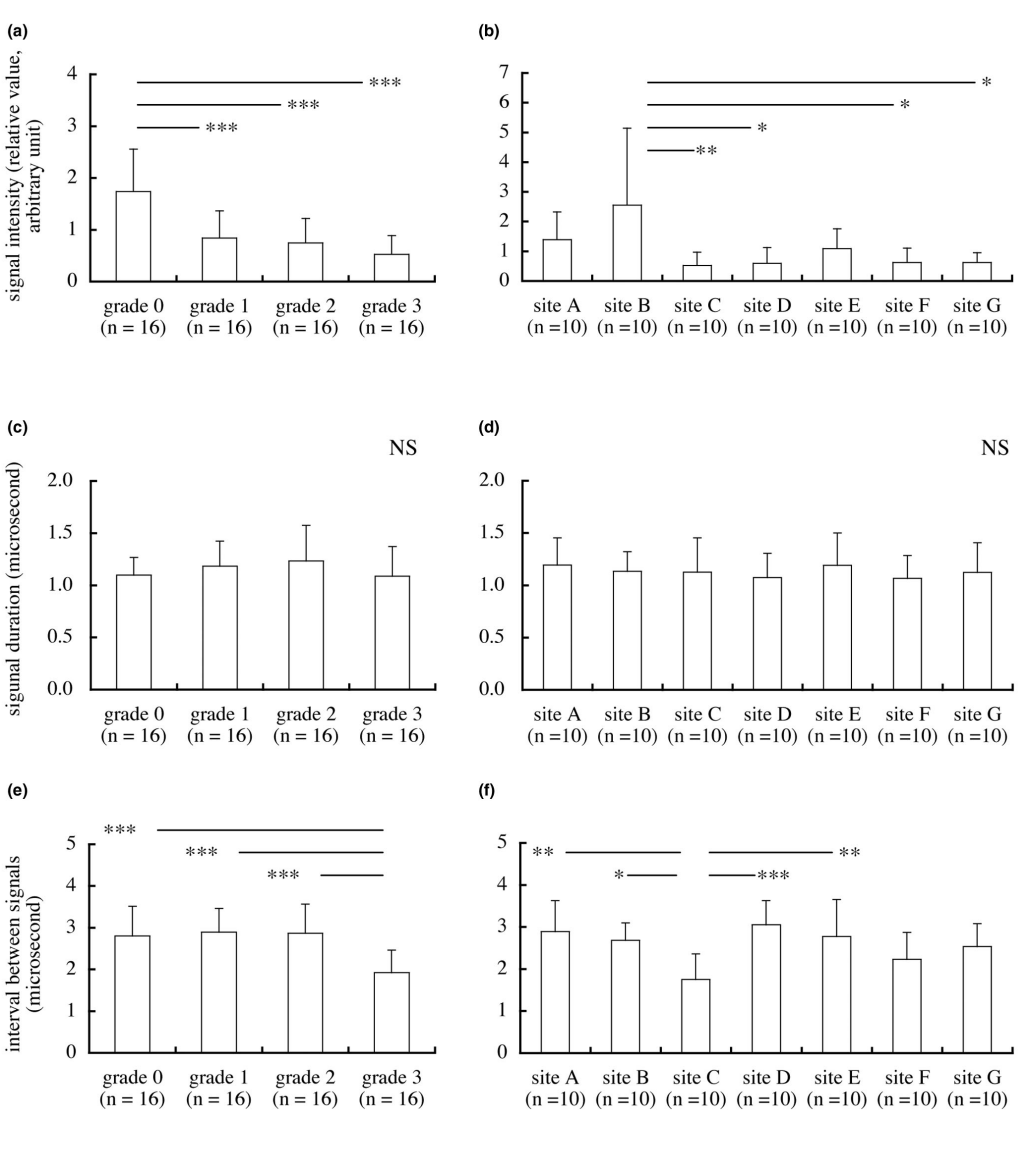
**Signal intensity, echo duration and interval between signals**. **(a) **The signal intensity (a measure of superficial cartilage integrity) of grade 0 cartilage was greater than the intensities of grade 1, grade 2 or grade 3 cartilage (mean and standard deviation). **(b) **The signal intensity at site B cartilage was significantly greater than the intensities at site C, site D, site F or site G cartilage. **(c) **No difference in echo duration (a parameter related to the surface irregularity) among the grades. **(d) **No difference in echo duration among the sites. **(e) **The interval between signals (that is, time of flight – which is related to thickness and ultrasound speed of cartilage) of grade 3 cartilage was less than the intervals of grade 0, grade 1 or grade 2 cartilage. **(f) **The interval between signals at site C was less than the intervals at site A, site B, site D or site E. *P < 0.05, **P < 0.01, ***P < 0.001; NS, not significant.

**Figure 4:**
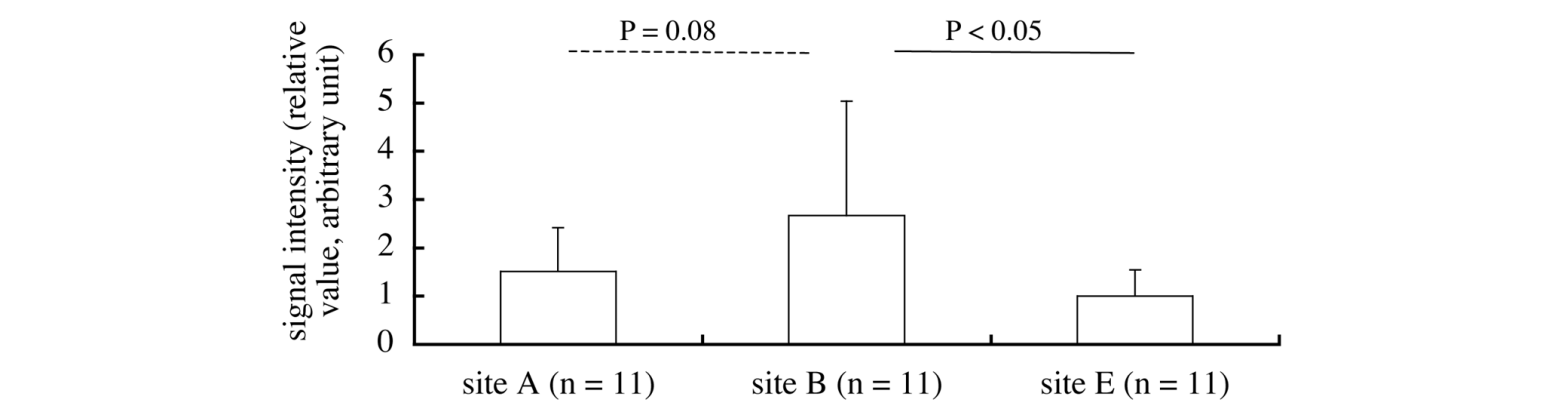
**Signal intensity of grade 0 cartilage**. The signal intensity of grade 0 cartilage at site B (femoral lateral condyle (posterior)) was significantly greater than that at site E (lateral tibial plateau (under the meniscus)), and tended to be greater than that at site A (femoral lateral condyle (anterior)).

The signal intensities (mean ± standard deviation, relative value, arbitrary units) of grade 0 (*n* = 16), grade 1 (*n* = 16), grade 2 (*n* = 16) and grade 3 (*n* = 16) cartilage were 1.74 ± 0.823 0.84 ± 0.525, 0.75 ± 0.471 and 0.53 ± 0.362, respectively (Figure 3a). The signal intensity of grade 0 cartilage was significantly greater than the intensities of grade 1, grade 2 or grade 3 cartilage (*P *< 0.001) (Figure 3a). The signal intensities at site A (*n* = 10), site B (*n* = 10), site C (*n* = 10), site D (*n* = 10), site E (*n* = 10), site F (n = 10) and site G (n = 10) were 1.39 ± 0.935, 2.56 ± 2.588, 0.52 ± 0.450, 0.59 ± 0.535, 1.08 ± 0.674, 0.63 ± 0.480 and 0.62 ± 0.330, respectively (Figure 3b). The signal intensity for site B cartilage was significantly greater than the intensities for site C (*P *< 0.01), site D (*P *< 0.05), site F (*P *< 0.05) and site G cartilage (*P *< 0.05) (Figure 3b). The signal intensities of grade 0 cartilage at site A (*n* = 11), site B (*n* = 11) and site E (*n* = 11) were 1.51 ± 0.905, 2.67 ± 2.369 and 1.00 ± 0.540, respectively; the signal intensity was greater at site B than at site E (*P *< 0.05) (Figure 4).

The echo durations of grade 0, grade 1, grade 2 and grade 3 cartilage were 1.10 ± 0.170 μs, 1.18 ± 0.242 μs, 1.23 ± 0.342 μs and 1.09 ± 0.283 μs, respectively (Figure 3c). Echo durations at site A, site B, site C, site D, site E, site F and site G were 1.19 ± 0.260 μs, 1.13 ± 0.188 μs, 1.13 ± 0.327 μs, 1.07 ± 0.233 μs, 1.19 ± 0.310 μs, 1.07 ± 0.217 μs and 1.12 ± 0.284 μs, respectively (Figure 3d). There was no difference in the echo duration among the grades and among the sites (Figure 3c,d).

The intervals between signals of grade 0, grade 1, grade 2 and grade 3 cartilage were 2.80 ± 0.715 μs, 2.89 ± 0.566 μs, 2.87 ± 0.700 μs, 1.92 ± 0.537 μs, respectively (Figure 3e). The interval for grade 3 cartilage was less than the intervals for grade 0, grade 1, or grade 2 cartilage (*P *< 0.001) (Figure 3e). The intervals between signals at site A, site B, site C, site D, site E, site F and site G were 2.89 ± 0.735 μs, 2.68 ± 0.416 μs, 1.76 ± 0.604 μs, 3.06 ± 0.575 μs, 2.77 ± 0.883 μs, 2.23 ± 0.638 μs and 2.54 ± 0.541 μs, respectively (Figure 3f). The interval between signals at site C was less than the intervals at site A (*P *< 0.01), site B (*P *< 0.05), site D (*P *< 0.001) and site E (*P *< 0.01) (Figure 3f).

The mean values for the signal intensity, the echo duration and the interval between signals for each site and for each ICRS grade of 20 knees are presented in Table 2.

**Table 2:** Signal intensity, echo duration and interval between signals at each site for each grade of cartilage from 20 knees

	Grade 0	Grade 1	Grade 2	Grade 3
Signal intensity (relative value, arbitrary units)				
Site A	1.51 ± 0.863	1.33 ± 0.775	1.05 ± 0.807	--
Site B	2.60 ± 1.945	0.96 ± 0.433	0.73 ± 0.502	--
Site C	--	--	0.82	0.57 ± 0.456
Site D	1.30	0.72 ± 0.751	0.52 ± 0.483	0.14
Site E	1.30 ± 0.788	0.37 ± 0.230	0.46	--
Site F	--	--	0.84 ± 0.329	0.54 ± 0.402
Site G	--	1.18 ± 0.218	0.63 ± 0.420	0.40 ± 0.169
Echo duration (*μ*s)				
Site A	1.05 ± 0.136	1.23 ± 0.224	1.34 ± 0.438	--
Site B	1.08 ± 0.201	1.28 ± 0.279	1.29 ± 0.392	--
Site C	--	--	1.10	1.11 ± 0.336
Site D	1.37	1.08 ± 0.301	1.29 ± 0.490	1.17
Site E	1.12 ± 0.220	1.34 ± 0.376	0.97	--
Site F	--	--	1.08 ± 0.083	1.04 ± 0.201
Site G	--	1.25 ± 0.220	1.09 ± 0.235	1.20 ± 0.389
Interval between signals (*μ*s)				
Site A	2.60 ± 0.694	3.07 ± 0.597	3.04 ± 0.672	--
Site B	2.79 ± 0.441	2.82 ± 0.428	2.72 ± 0.562	--
Site C	--	--	3.05	1.64 ± 0.504
Site D	3.90	3.27 ± 0.525	3.34 ± 0.681	2.39
Site E	2.77 ± 0.794	3.09 ± 0.747	3.68	--
Site F	--	--	2.71 ± 0.787	1.87 ± 0.647
Site G	--	1.79 ± 0.398	2.67 ± 0.619	2.49 ± 0.553

Data presented as mean ± standard deviation. For International Cartilage Repair Society grades, see Introduction. For anatomical location of sites, see Figure 1. The number of knees is shown in Additional file 1.

## Discussion

The present study shows the relationship between ICRS grades and ultrasound properties of articular cartilage. The signal intensity decreased with increasing ICRS grade (Figure 3a). Although differentiating between healthy cartilage and ICRS grade 1 cartilage may be difficult using mechanical testing alone [17], a differentiation could be detected using ultrasound. The ultrasound evaluation is performed within a very short time (<0.5 s) [20].

The signal intensity and the ICRS grade vary between sites within the knee. Indentation studies show that cartilage in the femoral condyles is stiffest, cartilage in the patellar surface of the femur is softer, and cartilage in the tibial plateau exposed by the menisci is softest [21,22]. In the present study, the signal intensity of grade 0 cartilage at site B was greater than that at site E (Figure 4). Cartilage at site B is located on the lateral condyle, and site E cartilage is located on the lateral tibial plateau exposed by the lateral meniscus (Figure 1). The data are therefore consistent with the two indentation studies [21,22]. Although the ultrasound technique differs from the indentation technique, the results are consistent with each other.

In the lateral condyle, however, the signal intensity of site A cartilage tended to be less than that of site B cartilage (*P *= 0.08) (Figure 4). Ultrasound reflection at the cartilage surface has been shown to be related to the integrity of the superficial cartilage [23,24]. There are therefore two possible interpretations of this observation. Because site A is located just anterior to site B, an early osteoarthritis event has occurred in the anterior cartilage of the lateral condyle and affected the signal intensity of site A cartilage. Alternatively, cartilage at site A originally has been more susceptible to deterioration than that at site B. We observed a greater percentage of osteoarthritis in site A cartilage than in site B cartilage. At site A, the grade 0, grade 1, grade 2 and grade 3 cartilage comprised 55% (11 out of 20 knees), 60%, 30% and 0%, respectively (Table 1). At site B, in contrast, the grade 0, grade 1, grade 2 and grade 3 cartilage comprised 80% (16 out of 20 knees), 40%, 25% and 0%, respectively (Table 1). These percentages suggest the incidence of early osteoarthritis in the lateral condyle may be higher in anterior cartilage (site A) than in posterior cartilage (site B).

Although the signal intensity of site E cartilage was less than that of site B cartilage, grade 0 cartilage at site E comprised 85% (17 out of 20 knees), which is greater than the percentage of grade 0 cartilage at site B (Table 1). Site E cartilage is located on the lateral tibial plateau exposed by the lateral meniscus. Site D cartilage is located on the central load-bearing region in the lateral tibial plateau. We observed that the medial meniscus was worn and very thin in most patients. In some patients, it had ruptured at the central part or the meniscus had disappeared completely. At sites F and site G, grade 0 cartilage was absent and grade 4 cartilage comprised a high percentage. The cartilage below the menisci was therefore protected from degeneration compared with the central load-bearing regions.

High-frequency pulse-echo ultrasound is sensitive for detecting degeneration of the superficial collagen-rich cartilage zone [10]. Ultrasound measurements appear to be related to changes in the extracellular matrix collagen and the fibrillar network organization [12]. Ultrasound can detect microstructural changes up to a depth of 500 *μ*m [18]. The signal intensity therefore provides information on the superficial collagen integrity of cartilage. The decrease in signal intensity in site C cartilage (Figure 3b) and the above site-specific differences in signal intensity suggest that the superficial collagen network was maintained in cartilage of the lateral condyle (site A and site B) but deteriorated in cartilage of the medial condyle (site C), in cartilage at the central load-bearing region in the lateral tibial plateau (site D) and in cartilage of the medial tibial plateau (site F and site G) in varus knee osteoarthritis.

In the present study, the percentages of the signal intensity of grade 1, grade 2 and grade 3 cartilage to grade 0 cartilage were 48% (0.84 versus 1.74), 43% (0.75 versus 1.74) and 30% (0.53 versus 1.74), respectively (Figure 3a). The interval between signals (a parameter of thickness) indicated that cartilage wear increased markedly from grade 2 to grade 3 (Figure 3e). The present study therefore suggests that a signal intensity <43% is indicative of cartilage degeneration. Although there was no distinctive difference in the intervals between signals for grade 1 cartilage and grade 2 cartilage (Figure 3e), surface recession and wearing of grade 2 cartilage was evident on macroscopic examination. A signal intensity <48% might therefore detect the surface recession of cartilage.

There was no difference in the echo duration among the grades. Because the low signal intensities of grade 1, grade 2 and grade 3 cartilage (48%, 43% and 30% of that of grade 0 cartilage, respectively) decreased earlier with a shorter time than that of grade 0 cartilage, detection of irregularity of grade 1, grade 2 and grade 3 cartilage using echo duration might be limited.

The interval between signals of grade 3 cartilage was significantly less than that of grade 0 cartilage (Figure 3e), but that of grade 1 cartilage and grade 2 cartilage did not differ from that of grade 0 cartilage. Although these data for grade 1 cartilage and grade 2 cartilage are not consistent with ICRS descriptions, the discrepancies can be explained by a decrease in the speed of sound in degraded cartilage [7,25,26]. The speed of sound is dependent on the cartilage water content, and an increase of water content induces the decrease of the speed of sound [25]. The water content increases with the swelling of the tissue [27-29]. Swelling in fibrillated cartilage [27] with superficial lesions, especially in grade 1 cartilage, occurs before significant cartilage loss – and probably arises from a reduction in the elastic restraint of the collagen network, allowing the glycosaminoglycans within the fibrillated tissue to swell to a greater degree of hydration [28]. Because the speed of sound is slightly lower in hydrated cartilage than in normal cartilage [25], the ultrasound value obtained from grade 1 cartilage may also reflect the slightly decreased speed of sound in the hydrated cartilage. The glycosaminoglycans in grade 2 cartilage, in which significant cartilage loss occurred, probably swell to a greater degree of hydration than those in grade 1 cartilage. The greater degree of hydration in the grade 2 cartilage affects the interval between signals. Information such as the macroscopic findings of cartilage degeneration is therefore helpful to interpret the interval between signals using a predefined speed of sound. An ultrasound arthroscopic probe (Figure 2a) may contribute to confirming visual findings in an area of questionable degeneration in very early stage of osteoarthritis.

## Conclusion

The ultrasound response of articular cartilage may be related to its ICRS grading. Ultrasound data indicate that the signal intensity decreases with increasing ICRS grade. Site-specific differences in signal intensity suggest that the superficial collagen network may be maintained in cartilage of the lateral condyle but may deteriorate in cartilage of the medial condyle and the medial tibial plateau in varus knee osteoarthritis. Ultrasound evaluation using the signal intensity – dependent on the ultrasound reflection coefficient at the cartilage surface – may be helpful to differentiate ICRS grades, especially grade 0 from grade 1 cartilage.

## Abbreviations

ICRS = International Cartilage Repair Society.

## Competing interests

The authors declare that they have no competing interests.

## Authors' contributions

HK, YN, MK, KY, YO, TS and KN participated in the ultrasound measurement during the surgery. HK and KM participated in the analysis of the ultrasound indices. HK and KY performed statistical analysis. YN conceived of the study and participated in its design and coordination. HK drafted the manuscript. YN and TN helped to draft the manuscript.

## Supplementary Material

Additional file 1 A file containing a table that presents the names of the knees and the number of different points measured at each site and at each grade. Click here for file
