# Supplementary material for: Ultrasound properties of articular cartilage in the tibio-femoral joint in knee osteoarthritis: relation to clinical assessment (International Cartilage Repair Society grade)
Source: Arthritis Res Ther. 2008 Jul 13;10(4):R78. doi: 10.1186/ar2452 (PMC2575624; doi:10.1186/ar2452)
Supplement: Additional file 1 — A file containing a table that presents the names of the knees and the number of different points measured at each site and at each grade. [file ar2452-S1.doc]

Appendix - Table A. Name of knees and number of different points measured at each site and each grade.

|  | **grade 0** | | **grade 1** | | **grade 2** | | **grade 3** | | **grade 4** | |
| --- | --- | --- | --- | --- | --- | --- | --- | --- | --- | --- |
|  | **NK (NPM)*** | | **NK (NPM)** | | **NK (NPM)** | | **NK (NPM)** | | **NK (NPM)** | |
| **site A** | **No. 2 (2)** | **No. 12 (6)** | **No. 1 (3)** | **No. 9 (2)** | **No. 1 (1)** |  |  |  | **No. 10 (1)** |  |
|  | **No. 3 (2)** | **No. 13 (2)** | **No. 2 (2)** | **No. 13 (1)** | **No. 4 (1)** |  |  |  | **No. 15 (1)** |  |
|  | **No. 8 (4)** | **No. 14 (4)** | **No. 4 (3)** | **No. 15 (2)** | **No. 6 (1)** |  |  |  | **No. 16 (2)** |  |
|  | **No. 9 (1)** | **No. 15 (3)** | **No. 5 (3)** | **No. 16 (2)** | **No. 7 (1)** |  |  |  | **No. 19 (1)** |  |
|  | **No. 10 (4)** | **No. 17 (5)** | **No. 6 (2)** | **No. 18 (1)** | **No. 9 (1)** |  |  |  |  |  |
|  | **No. 11 (4)** |  | **No. 7 (2)** | **No. 19 (2)** | **No. 18 (1)** |  |  |  |  |  |
| **site B** | **No. 1 (1)** | **No. 11 (4)** | **No. 1 (2)** |  | **No. 1 (1)** |  |  |  | **No. 3 (1)** |  |
|  | **No. 2 (3)** | **No. 12 (3)** | **No. 4 (3)** |  | **No. 5 (2)** |  |  |  | **No. 10 (1)** |  |
|  | **No. 3 (2)** | **No. 13 (4)** | **No. 5 (1)** |  | **No. 6 (2)** |  |  |  | **No. 19 (1)** |  |
|  | **No. 6 (2)** | **No. 14 (4)** | **No. 7 (2)** |  | **No. 15 (1)** |  |  |  |  |  |
|  | **No. 7 (1)** | **No. 15 (3)** | **No. 12 (1)** |  | **No. 16 (2)** |  |  |  |  |  |
|  | **No. 8 (4)** | **No. 17 (2)** | **No. 16 (2)** |  |  |  |  |  |  |  |
|  | **No. 9 (4)** | **No. 18 (2)** | **No. 17 (2)** |  |  |  |  |  |  |  |
|  | **No. 10 (4)** | **No. 19 (3)** | **No. 19 (1)** |  |  |  |  |  |  |  |
| **site C** |  |  |  |  | **No. 2 (1)** |  | **No. 1 (1)** | **No. 17 (4)** | **No. 1 (3)** | **No. 11 (1)** |
|  |  |  |  |  |  |  | **No. 2 (1)** | **No. 18 (1)** | **No. 2 (3)** | **No. 12 (4)** |
|  |  |  |  |  |  |  | **No. 3 (3)** | **No. 19 (1)** | **No. 3 (2)** | **No. 13 (2)** |
|  |  |  |  |  |  |  | **No. 4 (2)** |  | **No. 4 (2)** | **No. 14 (4)** |
|  |  |  |  |  |  |  | **No. 5 (1)** |  | **No. 5 (3)** | **No. 15 (4)** |
|  |  |  |  |  |  |  | **No. 8 (2)** |  | **No. 6 (2)** | **No. 16 (3)** |
|  |  |  |  |  |  |  | **No. 11 (3)** |  | **No. 7 (3)** | **No. 17 (2)** |
|  |  |  |  |  |  |  | **No. 13 (3)** |  | **No. 8 (3)** | **No. 18 (2)** |
|  |  |  |  |  |  |  | **No. 15 (2)** |  | **No. 9 (4)** | **No. 19 (4)** |
|  |  |  |  |  |  |  | **No. 16 (2)** |  | **No. 10 (4)** | **No. 20 (4)** |
| **site D** | **No. 9 (1)** |  | **No. 2 (1)** | **No. 11 (2)** | **No. 1 (3)** | **No. 13 (1)** | **No. 3 (1)** |  | **No. 13 (1)** |  |
|  |  |  | **No. 4 (2)** | **No. 12 (3)** | **No. 2 (2)** | **No. 15 (2)** |  |  | **No. 15 (1)** |  |
|  |  |  | **No. 5 (1)** | **No. 14 (3)** | **No. 4 (1)** | **No. 16 (2)** |  |  | **No. 19 (1)** |  |
|  |  |  | **No. 8 (3)** | **No. 17 (1)** | **No. 5 (2)** | **No. 17 (2)** |  |  |  |  |
|  |  |  | **No. 9 (2)** | **No. 18 (3)** | **No. 6 (2)** |  |  |  |  |  |
|  |  |  | **No. 10 (3)** |  | **No. 7 (1)** |  |  |  |  |  |
| **site E** | **No. 1 (3)** | **No. 10 (4)** | **No. 5 (1)** |  | **No. 19 (2)** |  |  |  | **No. 12 (1)** |  |
|  | **No. 2 (4)** | **No. 11 (4)** | **No. 6 (1)** |  |  |  |  |  |  |  |
|  | **No. 3 (3)** | **No. 12 (4)** | **No. 7 (2)** |  |  |  |  |  |  |  |
|  | **No. 4 (4)** | **No. 13 (3)** | **No. 15 (1)** |  |  |  |  |  |  |  |
|  | **No. 5 (3)** | **No. 14 (3)** | **No. 17 (1)** |  |  |  |  |  |  |  |
|  | **No. 6 (2)** | **No. 15 (2)** | **No. 18 (3)** |  |  |  |  |  |  |  |
|  | **No. 7 (1)** | **No. 16 (3)** | **No. 19 (1)** |  |  |  |  |  |  |  |
|  | **No. 8 (2)** | **No. 17 (2)** |  |  |  |  |  |  |  |  |
|  | **No. 9 (4)** |  |  |  |  |  |  |  |  |  |
| **site F** |  |  |  |  | **No. 3 (1)** |  | **No. 1 (2)** | **No. 13 (2)** | **No. 1 (1)** | **No. 10 (2)** |
|  |  |  |  |  | **No. 11 (1)** |  | **No. 2 (2)** | **No. 15 (3)** | **No. 2 (2)** | **No. 12 (3)** |
|  |  |  |  |  | **No. 17 (3)** |  | **No. 3 (1)** | **No. 16 (2)** | **No. 3 (1)** | **No. 13 (2)** |
|  |  |  |  |  |  |  | **No. 4 (1)** | **No. 17 (1)** | **No. 4 (1)** | **No. 14 (3)** |
|  |  |  |  |  |  |  | **No. 6 (1)** | **No. 18 (2)** | **No. 5 (2)** | **No. 15 (1)** |
|  |  |  |  |  |  |  | **No. 8 (2)** | **No. 19 (1)** | **No. 6 (2)** | **No. 16 (2)** |
|  |  |  |  |  |  |  | **No. 10 (2)** |  | **No. 7 (2)** | **No. 18 (1)** |
|  |  |  |  |  |  |  | **No. 11 (2)** |  | **No. 8 (2)** | **No. 19 (2)** |
|  |  |  |  |  |  |  | **No. 12 (1)** |  | **No. 9 (3)** | **No. 20 (3)** |
| **site G** |  |  | **No. 3 (1)** |  | **No. 2 (1)** | **No. 19 (1)** | **No. 4 (2)** |  | **No. 1 (2)** | **No. 12 (1)** |
|  |  |  | **No. 11 (2)** |  | **No. 3 (2)** |  | **No. 5 (1)** |  | **No. 2 (1)** | **No. 13 (1)** |
|  |  |  |  |  | **No. 8 (1)** |  | **No. 7 (1)** |  | **No. 3 (1)** | **No. 14 (2)** |
|  |  |  |  |  | **No. 9 (2)** |  | **No. 8 (2)** |  | **No. 4 (1)** | **No. 15 (3)** |
|  |  |  |  |  | **No. 10 (2)** |  | **No. 12 (1)** |  | **No. 5 (3)** | **No. 16 (2)** |
|  |  |  |  |  | **No. 11 (1)** |  | **No. 13 (2)** |  | **No. 6 (2)** | **No. 17 (3)** |
|  |  |  |  |  | **No. 12 (2)** |  | **No. 15 (1)** |  | **No. 7 (1)** | **No. 18 (1)** |
|  |  |  |  |  | **No. 13 (1)** |  | **No. 16 (2)** |  | **No. 9 (2)** | **No. 19 (2)** |
|  |  |  |  |  | **No. 14 (2)** |  | **No. 17 (1)** |  | **No. 10 (2)** | **No. 20 (1)** |
|  |  |  |  |  | **No. 18 (2)** |  | **No. 18 (1)** |  | **No. 11 (1)** |  |
|  | **NK (NPM)*: name of knees (number of points measured)** | | | | | | | |  |  |
